# Powerful Allies and Weak Consensus: Towards a Deeper Understanding of how Health-Harming Industries Seek to Influence Global Health Governance: Comment on "Competing Frames in Global Health Governance: An Analysis of Stakeholder Influence on the Political Declaration on Non-communicable Diseases"

**DOI:** 10.34172/ijhpm.2021.136

**Published:** 2021-09-21

**Authors:** Marco Zenone, Benjamin Hawkins

**Affiliations:** ^1^Faculty of Public Health & Policy, London School of Hygiene and Tropical Medicine, London, UK.; ^2^MRC Epidemiology Unit, School of Clinical Medicine, University of Cambridge, Cambridge, UK.

**Keywords:** Corporate Actors, Health-Harming Industries, Global Health, Global Health Governance, Corporate Influence

## Abstract

Suzuki et al have identified commonalities in the policy positions adopted at a global forum by commercial sector actors and high-income countries (HICs), on the one hand, and non-governmental organizations (NGOs) and low- and middle-income countries (LMICs), on the other, in ways that may allow commercial sector actors to block or delay evidence-based policies through the creation of political controversy. The ability of industry actors to draw on the support of the most politically and economically powerful countries for their favoured policy agenda is an important contribution to understanding the dynamics of global health governance in the area of non-communicable diseases (NCDs) and beyond. Here we assess the relevance of this paper for the field of corporate actors’ research and the potential avenues this opens up for further study. More specifically we emphasize the need for comparative, cross disciplinary research to examine the power of heath-harming industries and the relevance of these findings for decolonizing global health.

 Suzuki et al^1^ seek to capture the ways in which different actors sought to frame policy debates and shape the 2018 United Nations (UN) Political Declaration of the Third High-Level Meeting on the Prevention and Control of Non-Communicable Diseases (NCDs). They review 159 documents submitted by public and private stakeholders during the negotiation of the declaration. The authors determine that proposals to govern or introduce policy interventions on health-harming industries such as the alcohol, tobacco, or ultra-processed food and beverage industries – and their role as risk factors for NCDs – were widely opposed by private commercial actors and high-income countries (HIC). In contrast, low- and middle-income countries (LMICs) and non-governmental organizations (NGOs) advocated for evidence-based, cost-effective, population level interventions to reduce consumption of harmful products, such as taxing sugary drinks. Suzuki and colleagues found that when disagreement existed between opposing policy frames – for example on the regulation of health-harming industries – these were omitted from, or at least marginalised within the final declaration. The final version of the political declaration included those proposals over which there was no apparent opposition or opposing view, thus missing opportunities to advocate for the most effective policy measures and reduce the global burden of NCDs.

 The analysis provides several areas of meaningful reflection and potential action in global health. First, the involvement of private actors providing input on international health standards – and the apparent effectiveness of their attempts to frame policy outputs in line with their commercial priorities – emphasizes the importance of further opening the “black box” of health-harming industry practices in global forums to reveal the mechanisms of potential influence. To date, there are many examples of private actors using varying strategies such as lobbying, astroturfing, and producing and/or promoting flawed research to advance their favoured positions in international policy settings. In response to growing calls to reduce conflicts of interest in nutrition policy, the World Health Organization (WHO) developed a draft tool to manage interactions between country and private entities. Private actors argued that the WHO unfairly limits their engagement with global health entities while academics, public health advocates and some member-states argued that engagement with health-harming industries is incompatible with the Sustainable Development Goals.^2^ Health-harming industries seek to apply novel strategies to insert themselves into policy debates or decision-making forums. Consequently, appropriate mechanisms and processes are needed to protect public health from attempts to undermine global policies. Recently, the WHO Foundation, a new organization to elicit donors, was criticized for a “lack of clarity about the applicability of framework of engagement with non-state actors norms and practices” or addressing the possibility that the WHO could be viewed as “sacrific[ing] independence or impartiality to the commercial determinants of health in pursuit of funding.”^3^

 These efforts to protect health policy-making for corporate influence raise questions about the treatment of different industries and requires us to expand our understanding of what constitutes a health-harming industry. This includes challenging the logic of ‘tobacco exceptionalism’ which characterises the approach of many organisations and entities to engagement with corporate political actors. There is a need to expand the types of frameworks and approaches applied to the tobacco sector in such forums onto other health-harming industries (eg, alcohol and processed foods),^4^ while striving not to undermine the gains made in tobacco control. Similarly, technological advances in recent years mean that global health is now faced with a new range of potentially health-harming industries. For example, WHO and other global health institutions face emerging public health challenges such as the spread of misinformation on social media. Reflection the lessons learned from health-harming industries, the global health community may consider applying precautions engaging with social media corporations on addressing misinformation based on their previous failure to prevent, moderate, or act on misinformation, and the vested economic interests of social media corporations to HICs.

 Second, the role of health harming industries influencing global health declarations and other initiatives underscores the need for interdisciplinary input from different academic disciplines, non-commercial actors, civil society organizations, and public health to support the effective regulation of health harming industries and their products. When debating the implications of particular public health measures, such as nutrition labelling, the strength and voice of health-harming industries appear to outnumber the voice of public health and other public-health-related disciplines. For example, in the Codex Alimentarius Commission, a UN agency outlining food standards, Thow and colleagues identified greater representation of private actors compared to public health officials on front of package nutrition labelling discussions.^5^ Interdisciplinary perspectives can also contribute to a greater understanding of the commercial determinants of health, such as defining better measurement methods and conceptual understanding in empirical evaluations of industry activities on public health or associated risk factors.^6^ At present, much (though not all) of the research on the corporate determinants of health is conducted within the broad area of public health, using the approaches, assumptions and methods common to that. Consequently, more extensive use could be made of the theories, concepts and methods employed in closely related social science disciplines to bring additional analytical depth and insight. Different disciplines can start to bridge this gap by presenting or analyzing data in accessible formats, with consistency and clarity in the reporting of methods and limiting findings to claims justifiably derived from the data analyzed.

 Third, private sector actors, and trans-national corporations, exert power in many ways. McKee and Stuckler argue that health-harming industries display invisible power, characterized by strategies such as “defining the dominant narrative; setting the rules by which society, especially trade, operates; commodifying knowledge; and undermining political, social, and economic rights.”^7^ Lacy-Nichols and Marten point out that power can be exerted through both coercive and appeasing manners – in other words, power can be displayed evidently through intentional visible actions (eg, lawsuits) or through subtle actions through which industry actors seek to “neutralize” opposition to their favoured policy positions.^8^ This is particularly relevant in the development of global health standards. Industry actors may seek to appease critics of their involvement through voluntary codes of practise, self-regulation or corporate social responsibilities to promote perceived altruistic public health gestures to garner support for their engagement. Therefore, process-oriented analyses to capture corporate political strategies are necessary to protect global health organizations from potential conflicts of interest in their engagements with powerful private actors.

 Fourth, the power imbalance between HICs and LMICs appears to be reflected in the prioritisation of the economic interests and policy preferences of HICs in global health governance. The activities described by Suzuki and colleagues appear to reinforce state power imbalances and sidelining health protecting measures, such as front of package labelling, favoured by many LMICs governments facing obesity and NCD-related public health crises.^5^ To address the obvious imbalances between state actors in global health settings, future research is needed that engages with theories of power to understand the often subtle, indirect and hidden mechanisms through which this is exerted in policy-making settings.^8^

 Fifth, the apparent alignment of the positions advocated by private sector actors and HICs is worthy of reflection given the implications this has for advancing policy agendas. HICs, in which many of these global corporations are homed, tended to advocate for policy agendas which are amenable, but which were opposed by public health and other NGOs. This may simply be a reflection of the economic interest of these companies in their home markets and the extent to which ‘national champions’ have been able to influence national policy agendas and delegations to international forums and negotiations.^9^ The advocacy of economically powerful states is obviously a powerful tool in advancing sectoral interests and perspectives but this study reveals perhaps a more nuanced point about the dynamics of such negotiations. It underlines the structuring effect of uncertainty and doubt on policy debates in ways noted previously in the context of scientific debates on policy relevant evidence.^10^ What this study documents is a specific health-related case study of the potential power of fostering disagreement as a political strategy to avoid policy development in a global forum. Through the advocacy of specific policy positions and framing it appears possible to confine outputs to the lowest common denominator of politically uncontroversial measures. This mirrors finding from previous studies about the structuring and limiting effects of co-regulatory regimes on national NCD policy processes.^11^

 Finally, Suzuki and colleagues’ findings prompt us to reflect on the ongoing colonial characteristics of global health and the failure of HICs to look to, and learn from, LMICs public health practices and interests. As described by Büyüm et al, “[h]istories of slavery, redlining, environmental racism and the predatory nature of capitalism underpin the design of global and public health systems, resulting in structural, racial and ethnic inequities within Black, Indigenous and People of Color (BIPOC) communities globally.”^12^ Colonial thinking patterns have led to institutions in HICs viewed as more prestigious and valued than those in LMICs and led to problematic notions of superiority in public health initiative leadership.^13^ The failure of HICs to learn from LMICs is prominently displayed during the coronavirus disease 2019 (COVID-19) pandemic. Leadership from LMICs such as Vietnam has emerged highly effectively – quickly adopting evidence-based containment strategies, such as investing in testing, travel screening, sharing accurate information, and contact tracing.^14^ In contrast, HICs such as the United Kingdom failed to learn from the example of Asian states more immediately effected by the pandemic in developing their strategic responses to the pandemic. In the area of NCDs too, policy innovations by LMICs have failed to lead to policy transfer in HIC setting and has at times led to opposition instead of learning.^15^

 In summary, Suzuki and colleagues’ analysis provides the opportunity for a moment of critical reflection within the global health research and practice communities. The role of health-harming industries in influencing international health standards, with the support of powerful allies in HICs, represents a challenge for global health. To address the current situation, interdisciplinary research and organization is needed. Particular focus is needed to examine the interactions between state and non-state actors with public health consequences; to increase transparency and bolster conflict of interest procedures; and to advance the process of decolonizing global health.

## Ethical issues

 Not applicable.

## Competing interests

 Authors declare that they have no competing interests.

## Authors’ contributions

 MZ and BH both contributed to manuscript conceptual design, writing, and editing.
